# *Enterococcus durans* SL70, a Novel Exopolysaccharide Producer from Traditional Sourdough Fermentation of Einkorn (*Triticum monococcum* L. ssp. *monococcum*)

**DOI:** 10.17113/ftb.62.04.24.8606

**Published:** 2024-12

**Authors:** Berna Genc, Seyda Merve Karatas, Merve Tuğçe Tunç

**Affiliations:** 1 Department of Genetics and Bioengineering, Gumushane University, Baglarbasi Road, 29100 Gumushane, Turkey; 2 Department of Food Engineering, Gumushane University, Baglarbasi Road, 29100 Gumushane, Turkey

**Keywords:** einkorn, *Enterococcus durans*, exopolysaccharide, lactic acid bacteria, MALDI-TOF MS, Siyez

## Abstract

**Research background:**

Given the potential of microbial exopolysaccharides from lactic acid bacteria in various industrial processes, alternative sources for the isolation of lactic acid bacteria are highly topical. In this study, we used a traditional sourdough from einkorn (*Triticum monococcum* L. ssp. *monococcum*) as a source of lactic acid bacteria for the isolation, identification and determination of exopolysaccharide producers.

**Experimental approach:**

The sourdough was prepared from einkorn according to the traditional method. Lactic acid bacteria were isolated and purified using the single colony technique on MRS and M17 media. The isolates were characterised using matrix-assisted laser desorption ionization-time of flight mass (MALDI-TOF) spectrometry. All isolates were analysed for extracellular polysaccharide production and one isolate was selected for purification and characterisation of its polysaccharide.

**Results and conclusions:**

The isolates were identified as *Lactobacillus plantarum*, *L. paraplantarum*, *L. brevis*, *Pediococcus pentosaceus*, *Enterococcus faecium* and *E. durans*. The production of exopolysaccharides by all lactic acid bacteria was evaluated and it was found that all strains (except one) were capable of producing exopolysaccharides. One polysaccharide (EPS-SL70) was purified from the isolates of *E. durans* SL70. This anionic heteropolysaccharide had, in addition to the carbohydrate backbone, a protein structure that did not contain nucleic acid. The carbohydrate backbone consisted of mannose, glucose, rhamnose, arabinose, xylose and galactose.

**Novelty and scientific contribution:**

The microbial flora of traditional einkorn sourdough has been identified in this study and represents the first report on the exopolysaccharide production by lactic acid bacteria in traditional einkorn sourdough. Additionally, *Enterococcus durans* from einkorn sourdough was identified as a new exopolysaccharide producer.

## INTRODUCTION

Einkorn (*Triticum monococcum *L*.* ssp. *monococcum*) also known in Turkey as Siyez, is a widely recognised cereal variety. This diploid species is particularly rich in fibre and essential nutrients such as vitamins and minerals, like iron, phytosterol, lutein, B1, B2 and B6, which have higher bioavailability (*1*, *2*). The sugar composition of einkorn consists of sucrose, glucose, fructose and maltose, with a total sugar mass fraction of 26.7 g/kg. The folic acid mass fraction in einkorn is between 429 and 678 mg/kg, while other important values include 11.6 g/100 g moisture, 65 g/100 g carbohydrates and 11.83–25.2 g/100 g protein (*3*, *4*). Zrcková *et al.* (*5*) found that einkorn has on dry mass basis a higher total polyphenol content (744.97 mg/kg) than wheat (702.15 mg/kg). Additionally, einkorn is characterised by a high content of flavonoids, which is 3.8 times higher than that of emmer wheat (*6*). The tocol mass fraction in einkorn is between 19.6 and 109.89 µg/g, exceeding that of bread wheat (23.3–79.7 µg/g) (*7*).

Lactic acid bacteria (LAB) represent an important group of bacteria with crucial industrial applications. These bacteria, characterised as Gram-positive, catalase-negative, facultatively anaerobic, non-motile, rod- or coccus-shaped bacteria, have a long history of safe use in food production due to their beneficial effects on human health and fermentation. LAB play a vital role in various sectors, including food production, treatment of diseases and production of macromolecules, enzymes and metabolic substances (*8*). Their antimicrobial properties and production of metabolites make them valuable for food biocontrol. In regions like the European Union (EU) countries, where the use of stabilisers is restricted, thickening cultures containing LAB are commonly used. LAB can produce various polysaccharides and products containing these bacteria are commercially available for thickening purposes in the EU and the USA (*9*). Furthermore, LAB are known for their texturising properties and are naturally present in numerous fermented products.

Microbial polysaccharides are biopolymers produced by microorganisms as a byproduct of their metabolic processes. These polysaccharides can be either water-soluble or insoluble and can be categorised as ionic or non-ionic carbohydrate-based compounds (*10*). They are typically classified into three main groups: endopolysaccharides (found within the cell), capsular polysaccharides (attached to the cell surface) and exopolysaccharides (released into the extracellular environment) (*11*, *12*). These polysaccharides have different structural properties based on factors such as the composition of monosaccharides, electrical charge, bonding patterns, side chains, chain length and branching frequency. In contrast to plant gums such as locust bean and gum arabic, microbial polysaccharides show higher water solubility and stability in a wide range of environmental conditions including temperature, pH and ionic strength. These polysaccharides, which are not used as energy sources, play crucial roles in reducing water activity, defending against phage attacks and phagocytosis, protecting against toxic compounds and antibiotics, managing osmotic pressure and facilitating the formation of biofilms that aid in cell recognition, adhesion to surfaces and colonisation of different ecosystems (*13*).

Polymerase chain reaction (PCR) and other PCR-based methods are molecular techniques that provide precise and accurate results for the identification of organisms at the species and sub-species level. However, these methods are costly, time-consuming and labour-intensive, making them unsuitable for routine identification (*14*). Mass spectrometry (MS) has been used to identify bacteria for many years. Anhalt and Fenselau (*15*) were the first to propose its use. Over time, the MS technique has been improved by incorporating different structural components of bacterial cells. Recently, Holland *et al.* (*16*) have shown that MALDI-TOF can be used to identify bacteria without the need for pretreatment of the cells. This innovative approach has proven effective in identifying bacteria at both the genus and species level (*17*-*19*).

In recent years, research has focused largely on novel microbial polysaccharides as they are widely used in industries such as food, textiles, detergent, cosmetics, microbial enhanced petroleum remediation, agriculture and wastewater treatment. These polysaccharides have various functions including gelling, thickening, adhesive, biofilm-forming, anti-tumour, antiviral and anti-inflammatory properties due to their distinct physicochemical and rheological properties. Einkorn wheat, a highly nutritious food with a low glycaemic index, is gaining increasing attention due to its unique properties compared to other flours like white flour, leading to its increasing use in the food industry. Despite the growing interest in einkorn, there is only a few studies on the production of sourdough from this particular wheat variety (*20*).

The aim of this study is to produce einkorn sourdough in traditional einkorn yeast fermentation, isolate and identify lactic acid bacteria that are able to produce exopolysaccharides from einkorn sourdough for the first time, and determine the production of new polysaccharides from sourdough by *Enterococcus durans*.

## MATERIALS AND METHODS

### Sourdough fermentation process

Einkorn flour (1.75 % fat, 64.9 % carbohydrate, 9.7 % fibre and 9.6 % protein; Dogalsan, Ankara, Turkey) was used as material and all the chemicals were purchased from Sigma-Aldrich, Merck (St. Louis, MO, USA), unless otherwise stated. A mixture of 100 g of flour and 100 mL of tap water was prepared and allowed to ferment at 36 °C for 120 h to obtain traditional einkorn sourdough. After fermentation, a fresh mixture of water and flour was inoculated with the matured sourdough from the previous day in a 1:1 ratio, with five daily replenishments (*21*).

### Isolation and identification of microbiota

Serial dilutions were prepared in 0.85 % NaCl solutions up to a dilution of 10^-8^ to isolate pure colonies. Subsequently, 0.1 mL of each dilution was spread on de Man, Rogosa and Sharpe (MRS) (Merck Milipore, Darmstadt, Germany) and M17 (Merck Milipore) agar plates for lactic acid bacteria and potato dextrose agar (PDA) for yeasts. The plates were then placed in anaerobic jars (Merck Millipore) containing Anaerocoult® A (Merck Millipore) and incubated at 36 °C for 72 h. Isolates were purified based on colony characteristics and selected colonies were stored at -86 °C in a medium containing 15 % glycerol. The isolates were identified using conventional methods such as Gram staining and catalase tests. Additionally, the matrix-assisted laser desorption ionization-time of flight mass spectrometry (MALDI-TOF MS; Bruker Daltonics GmbH, Bremen, Germany) technique was used to determine the genus and species of the isolates at Mustafa Kemal University (Centre for Implementation and Research of Plant Health Clinic, Hatay, Turkey). Protein was extracted from bacterial isolates on MRS and M17 agar plates by formic acid method (*22*). Protein spectra were obtained by MALDI-TOF Biotyper and then compared with the protein spectra of reference bacterial isolates in the microbial library of the device using MALDI Biotyper Real-Time Classification (RTC) software (Biotyper 3.0; Microflex LT; Bruker Daltonics GmbH).

### Cultivation and polysaccharide extraction

MRS and M17 broth were used for cultivation and production of polysaccharides by lactic acid bacteria. The bacterial isolates were cultured in 10 mL of MRS and M17 broth with a 1 % inoculum and then incubated at 36 °C for 72 h. After fermentation, the biomass was separated and then trichloroacetic acid (Isolab Laborgeräte GmbH, Weinheim, Germany) was added to a final concentration of 4 % (*m*/*V*) to remove contaminants. Chilled ethanol was added at three times the supernatant volume to precipitate the polysaccharide at 4 °C overnight (*23*). Centrifugation (Allegra X30; Beckman Coulter, Brea, CA, USA) at 3900×*g* for 15 min was performed after each step to separate biomass, impurities and polysaccharide. The partially purified polysaccharide sample was then lyophilised for further analysis.

### Determination of total sugar and protein content

Different solvents were used to determine the solubility of the polysaccharide and it was soluble only in water. The total sugar content was quantified using the phenol-sulfur method (*24*) with glucose as standard. The total protein content was determined using the Bradford method with bovine serum albumin as standard (*25*), and the spectrum was scanned in the range of 200−1000 nm.

### Elemental analysis

The elemental composition was analysed with an elemental analyser (Truespec Micro; Leco, St. Joseph, MI, USA) to determine the content of nitrogen, carbon, hydrogen and oxygen in the sample.

#### Fourier-transform infrared spectroscopy

A mass of 2 mg of polysaccharide sample was mixed with 100 mg of potassium bromide, crushed and placed in a 1-mm pellet for a Fourier-transform infrared (FTIR) spectrum analysis equipped with Spectrum software v. 10.5.2 (PerkinElmer Spectrum 3, Shelton, CT, USA). The spectra were recorded in a frequency range of 400–4000 cm^−1^.

### Monosaccharide composition

The composition of monosaccharides was analysed using gas chromatography-mass spectrometry (GC-MS model 7890A; Agilent Technologies, Santa Clara, CA, USA) by the breakdown of polysaccharide structures and the subsequent volatilisation of the monosaccharides (*26*). Glucose, fructose, mannose, xylose, rhamnose and arabinose were used as standards and a similar methodology was applied, except for acid hydrolysis.

### Quantification of zeta potential

Zeta potential of the polysaccharide was measured at 25 °C using dynamic light scattering analysis. Malvern Zetasizer Nano Zsp (Malvern, UK) was used for this measurement.

### X-ray diffraction analysis

A polysaccharide sample of 100 mg was analysed using the SmartLab XRD instrument (Rigaku, Tokyo, Japan) with a scanning speed of 2°/min in an angular span from 30° to 100°.

### Cytotoxicity of polysaccharides

Cytotoxicity was evaluated at the Drug Administration and Research Center at Istanbul Bezmialem University, Turkey. To assess cell viability, the MTT assay was carried out on the CCD-1079Sk fibroblast cell line at a cell count of 10^4^ cell/well. The cells were cultured at 37 °C with 5 % CO_2_ for 24 h. A control group was maintained in DMEM-F12 supplemented with 10 % fetal bovine serum (FBS) and 1 % penicillin/streptomycin (*27*).

## RESULTS AND DISCUSSION

### Microbial composition of einkorn sourdough

The data in Table 1 show the values obtained in the production of einkorn sourdough. The cell counts of yeast and lactic acid bacteria (LAB) were determined both before and after the fermentation and showed that sourdough production was successful after a 10-day fermentation. After this period, the intrinsic microbial content of the einkorn was found to increase approx. 8-fold for the LAB and 2-fold for the yeasts, resulting in a total lactic acid bacteria and yeast cell counts of 8.32 and 4.08 log (CFU/g), respectively. Previous studies by Lim *et al.* (*28*) found LAB cell counts of 8.0–9.3 log (CFU/g) post-sourdough production, while Moroni *et al.* (*29*) reported yeast cell counts of 3.0–7.3 log (CFU/g) and lactic acid bacteria cell counts of 9.0–9.9 log (CFU/g).

**Table 1:** Changes in microbial load during sourdough production from einkorn flour

	**Before fermentation**			**After fermentation**		
**Total**	*N*(colony)	DF	*N*/(log CFU/g)	*N*(colony)	DF	*N*/(log CFU/g)
**Yeast**	2	10**^-2^**	2.30	12	10**^-3^**	4.08
**LAB**	2	10**^-1^**	1.30	21	10**^-7^**	8.32

DF=dilution factor, LAB=lactic acid bacteria

The composition of the microbial community in einkorn sourdough is shown in Table S1, Table S2 and Table S3. As a result of the MALDI-TOF analysis, log score or match values were obtained for each isolate. These values ranged from 0 to 3 and each value indicated that the isolate was similar to specific bacteria in the library based on data from the NCBI database (*30*). The successful production of sourdough from einkorn was confirmed by analysing 22 isolates, of which 16 were *Saccharomyces cerevisiae* (70 %) and 6 were *Candida lusitaniae* (30 %) (Table S1). In addition, among the 56 isolates obtained after LAB isolation (Table S2 and Table S3), 43 were *Lactobacillus plantarum* (75 %), 5 were *L. paraplantarum* (8.7 %), 4 were *L. brevis* (7 %), 2 were *Pediococcus pentosaceus* (3.4 %), 1 was *Enterococcus faecium* (1.8 %) and 1 was *E. durans* (1.8 %). Saeed *et al.* (*31*) emphasised the dominance of *L. brevis* and *L. plantarum* as important lactic acid bacteria for sourdough production, while *S. cerevisiae* was the only species required for sourdough production. Ferraz *et al.* (*32*) reported that the quality of sourdough was improved by the co-fermentation of *L. plantarum* and *S. cerevisiae*, but the lactic acid bacteria had a direct effect on acidification and the final product. Cakır *et al.* (*20*) isolated lactic acid bacteria from einkorn sourdough and reported that the dominant lactic acid bacteria belonged to the species *P. pentosaceus*, *L. brevis*, *L. paraplantarum* and *L. plantarum*. Wieser *et al.* (*33*) reported that *L. plantarum* and *E. faecalis* played a joint role in sourdough production and were effective in gluten proteolysis, in contrast to the non-proteolytic *L. plantarum* and *E. faecalis*.

### Extracellular polysaccharide synthesis by lactic acid bacteria

The study investigated the polysaccharide production of lactic acid bacteria obtained from einkorn sourdough. The results, expressed as dry mass in Table S4, showed that exopolysaccharide concentrations varied between 0.4 and 2.4 g/L. Only one strain (SL-1) did not produce any polysaccharides. In contrast, all other strains, including *L. plantarum*, *L. paraplantarum*, *L. brevis*, *P. pentosaceus*, *E. faecium* and *E. durans* showed the ability to produce exopolysaccharides. Liu *et al.* (*34*) identified *Lactobacillus* spp. and *L. plantarum* as exopolysaccharide producers isolated from traditional sourdough.

Numerous studies have also shown that different strains of *Lactobacillus* spp. and other lactic acid bacteria found in different types of sourdough have the ability to produce polysaccharides (*35*, *36*). Abedfar *et al.* (*37*) isolated lactic acid bacteria from wheat bran sourdough and identified *L. plantarum* and *P. pentosaceus* as the predominant species. The production of polysaccharides by *P. pentosaceus* was 0.26 g/L and by *L. plantarum* 0.4 g/L. Ogunsakin *et al.* (*38*) identified *P. pentosaceus* SA8 and *P. pentosaceus* LD7 as producers of polysaccharides. Manini *et al.* (*39*) reported that *L. plantarum*, *L. brevis* and *P. pentosaceus* were able to produce exopolysaccharides in sourdough when cultivated on different carbon sources such as glucose, sucrose, raffinose, maltose, lactose and starch. Ispirli *et al.* (*40*) isolated and characterised *E. durans* as a polysaccharide producer from koumiss and kurut. Additionally, Jung *et al.* (*41*) identified *E. faecium* as a polysaccharide producer in sourdough.

### Polysaccharide characterisation

**Fig. S1:**
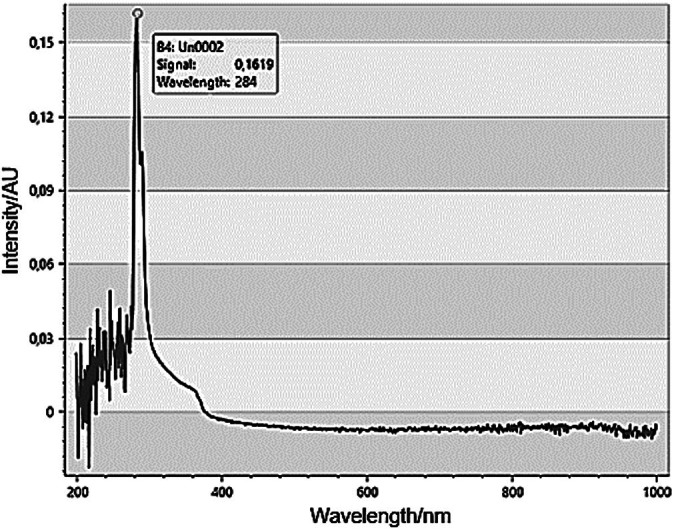
Spectrum of the exopolysaccharide EPS-SL70

The primary organism identified among the isolates was *E. durans* SL70, which produced extracellular polysaccharides when isolated from einkorn sourdough. The exopolysaccharide from *E. durans* SL70 in M17 medium, designated EPS-SL70, had a total carbohydrate concentration of (3.7±0.3) g/L and a protein concentration of 0.12 g/L. The absence of a peak at 260 nm indicates the absence of nucleic acid (Fig. S1). In a study by Vosough *et al.* (*42*), *Enterococcus* spp. were identified in Iranian kishk, and the carbohydrate and protein concentrations of EPS from *E. durans* K48 ranged from 0.76 to 2.39 g/L and 0.2 to 0.52 mg/L, respectively.

Elemental analysis showed that EPS-SL70 contained carbon (30.2 %), hydrogen (5.2 %) and nitrogen (3.6 %). Zanzan *et al.* (*43*) identified a polysaccharide from *E. faecium* F58 with a carbon content of 44.27 %. Gu *et al.* (*44*) reported that the polysaccharide derived from *E. durans* consisted of carbon (41.08 %) and hydrogen (7.23 %), without nitrogen, suggesting the presence of protein.

**Fig. 1:**
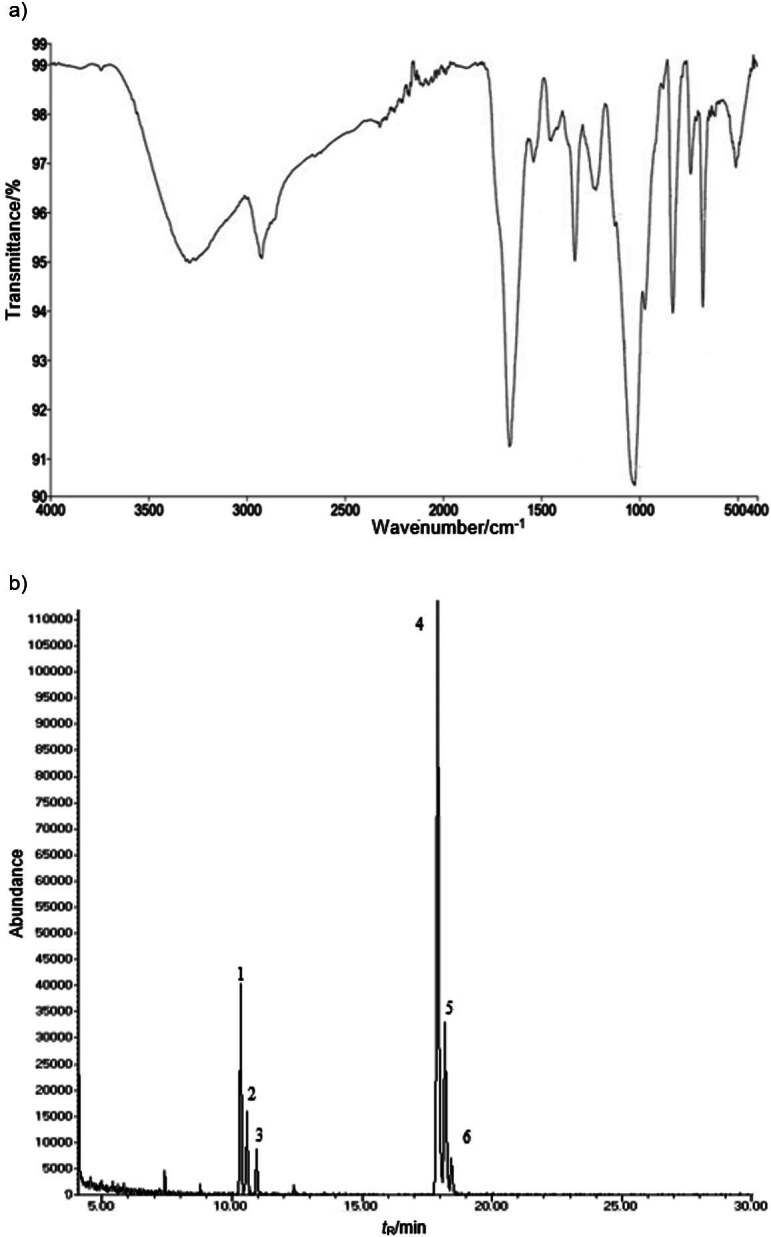
The results of: a) FTIR spectrum of *E. durans* SL70 polysaccharide and b) GC-MS spectra of monosaccharides: 1=rhamnose (10.3 min), 2=arabinose (10.6 min), 3=xylose (11 min), 4=mannose (17.9 min), 5=glucose (18.1 min) and 6=galactose (18.4 min)

FTIR analysis revealed ten distinct bands at specific frequencies. Fig. 1a shows a band profile reminiscent of common polysaccharide structures (*42*, *45*, *46*). The frequency at 3280 cm^-1^ (79.3 %) was attributed to intracellular hydrogen bonds or hydroxyl groups, while the frequency at 1657 cm^-1^ (52.6 %) indicated C=O stretches of amide bonds, confirming the presence of a protein structure within the carbohydrate backbone of the extracellular polysaccharide. The vibration at 1535 cm^-1^ (78.3 %) was associated with the carboxyl or carboxylate group. Additionally, the frequencies at 1329 cm^-1^ (67.2 %) and 1223 cm^-1^ (75.7 %) were associated with C-H stretching deformation. The presence of C–C vibration in the pyranose form of sugars was identified at 1123 cm^-1^ (80.8 %) and 1045 cm^-1^ (73.8 %). Furthermore, frequencies at 830 cm^-1^ (59.2 %), 736 cm^-1^ (76.4 %) and 675 cm^-1^ (58.1 %) indicated configurations (α and β) in the pyranose form, α-glucans and the carbohydrate skeletal vibrations, respectively.

The analysis of monosaccharide composition of the polysaccharide revealed that EPS-SL70 was a heteropolysaccharide composed mainly of mannose (60 %), glucose (15 %), rhamnose (13 %), arabinose (5 %), xylose (3 %) and galactose (3 %), as shown in Fig. 1b. *E. durans* K48 also produced a polysaccharide with a similar structure, but with a higher amount of galactose (*42*). *Lactobacillus* spp. showed the ability to produce polysaccharides containing rhamnose, glucose, galactose and mannose units (*47*). Similarly, *Leuconostoc pseudomesenteroides* RJ-5 produced extracellular polysaccharides containing mannose, glucose, arabinose, xylose and galactose (*48*).

The zeta potential, mobility and conductivity of EPS-SL70 were (-15.2±0.6) mV, (-1.2±0.05) cm^2^/(V∙s) and (1.42±0.08) mS/cm, respectively. The low value of zeta potential was indicative of the negative charge of the polysaccharide backbone. It was concluded that EPS-SL70 is an anionic heteropolysaccharide. *S. thermophilus* CRL1190 had a cytoprotective polysaccharide with a zeta potential of (-5.4±0.9) mV (*49*). The polysaccharide kefiran, produced from kefir grains using UHT skimmed milk, was negatively charged in different aqueous solutions and at different pH values (*50*).

**Fig. 2:**
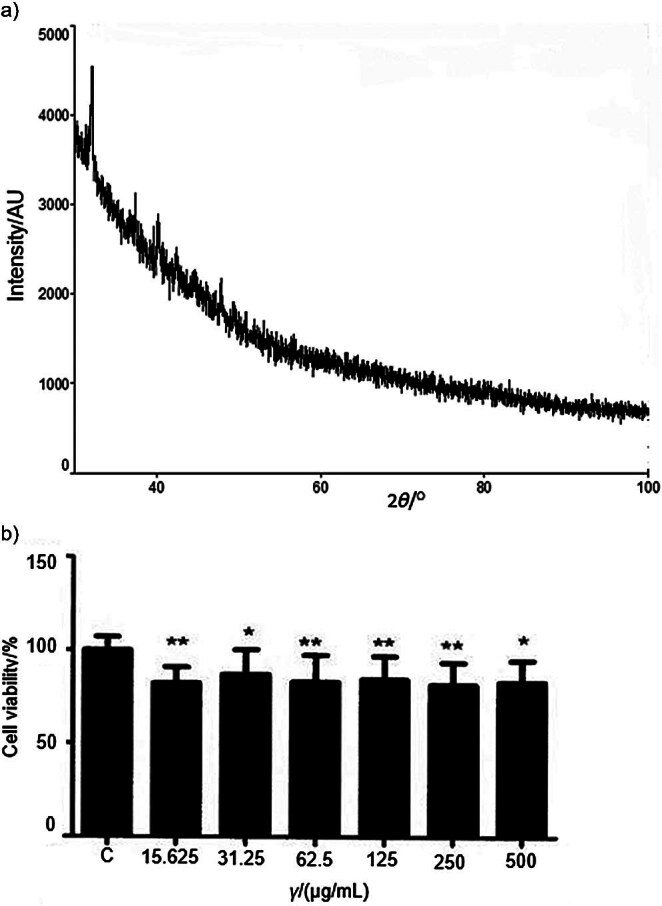
XRD pattern (a) and cytotoxicity effect (b) of EPS-SL70 on human fibroblast cell line (CCD-1079Sk) (*p<0.05 and **p<0.01). C=control

X-ray diffraction, the most widely used technique for the determination of the crystalline or non-crystalline (amorphous) nature of a polymer, was performed to estimate the phase identification of EPS-SL70 and the narrow peaks showed crystalline form (*51*). EPS-SL70 was found to have a uniform structure with a large extended peak that represents strong crystallinity without amorphous regions (Fig. 2a). The polysaccharides obtained from *E. faecium* MC-5 (*51*) and *Lactobacillus* sp. had the same structure (*52*)[REMOVED HYPERLINK FIELD].

Fibroblasts are the central cells of connective tissue and fibroblast dysfunction causes many defects in this tissue. They are affected by various substances and their activity can decrease, leading to connective tissue defects. In this study, a normal human fibroblast cell line (CCD-1079Sk) was used to evaluate the toxicity of EPS-SL70 (Fig. 2b), and no significant effect was observed at all concentrations. According to Hala *et al.* (*53*), lactic acid bacteria were determined to be safe when the survival rate was more than 80 %. Therefore, the cytotoxicity of the polysaccharide from *E. durans* SL70 can be neglected (p<0.05 and p<0.01).

## CONCLUSIONS

The ability of lactic acid bacteria to produce exopolysaccharides in einkorn sourdough was determined for the first time in this study. The results showed that einkorn and sourdough contain many different types of microorganisms. Lactic acid bacteria capable of producing polysaccharides can cooperate with yeasts in the formation of sourdough by releasing their polysaccharide. Therefore, a possible synergistic interaction between bacteria and yeasts in einkorn sourdough could be investigated in the future. The fact that the polysaccharide is not cytotoxic to human fibroblast cell lines makes it a candidate for use in different industrial applications, especially for products for human consumption. The characterisation of the polysaccharide provides important and preliminary information about its structure, but advanced techniques can be selected depending on the industrial application.

## Appendix

**Table S1:** MALDI-TOF mass spectrometry analysis of the isolates of yeast obtained from sourdough of einkorn flour

**Isolate**	Similarity	Biotyper log(score)	NCBI identifier
**SM-1**	*Saccharomyces cerevisiae*	2.025	4932
**SM-2**	*Saccharomyces cerevisiae*	2.230	4932
**SM-3**	*Saccharomyces cerevisiae*	2.034	4932
**SM-4**	*Saccharomyces cerevisiae*	2.010	4932
**SM-5**	*Saccharomyces cerevisiae*	1.928	4932
**SM-6**	*Saccharomyces cerevisiae*	1.786	4932
**SM-7**	*Saccharomyces cerevisiae*	1.994	4932
**SM-8**	*Candida lusitaniae*	2.209	3691
**SM-9**	*Saccharomyces cerevisiae*	1.957	4932
**SM-10**	*Saccharomyces cerevisiae*	1.895	4932
**SM-11**	*Saccharomyces cerevisiae*	2.111	4932
**SM-12**	*Saccharomyces cerevisiae*	1.933	4932
**SM-13**	*Saccharomyces cerevisiae*	1.839	4932
**SM-14**	*Saccharomyces cerevisiae*	2.103	4932
**SM-15**	*Candida lusitaniae*	2.244	3691
**SM-16**	*Candida lusitaniae*	2.211	3691
**SM-17**	*Candida lusitaniae*	2.213	3691
**SM-18**	*Candida lusitaniae*	2.043	3691
**SM-19**	*Candida lusitaniae*	2.343	3691
**SM-20**	*Saccharomyces cerevisiae*	2.139	4932
**SM-22**	*Saccharomyces cerevisiae*	2.163	4932
**SM-23**	*Candida lusitaniae*	2.187	3691

Biotyper log(score) value: 3.0–2.3=highly probable species identification, 2.299 –2.000=definite genus identification, probable species identification, 1.999–1.700=probable genus identification, 1.699–0.0=not reliable identification. NCBI=The National Center for Biotechnology Information (*30*)

**Table S2:** MALDI-TOF mass spectrometry analysis of isolates of lactic acid bacteria grown on MRS medium obtained from sourdough of einkorn flour

**Isolate**	Similarity	Biotyper log(score)	NCBI identifier
**SL-1**	*Lactobacillus plantarum*	2.341	1590
**SL-2**	*Lactobacillus plantarum*	2.301	1590
**SL-3**	*Lactobacillus plantarum*	2.340	1590
**SL-4**	*Lactobacillus plantarum*	2.350	1590
**SL-5**	*Lactobacillus plantarum*	2.455	1590
**SL-6**	*Lactobacillus plantarum*	2.442	1590
**SL-7**	*Pediococcus pentosaceus*	2.085	1255
**SL-8**	*Lactobacillus plantarum*	2.335	1590
**SL-9**	*Lactobacillus plantarum*	2.355	1590
**SL-10**	*Lactobacillus plantarum*	2.456	1590
**SL-11**	*Lactobacillus plantarum*	2.422	1590
**SL-12**	*Lactobacillus plantarum*	2.450	1590
**SL-13**	*Lactobacillus plantarum*	2.393	1590
**SL-14**	*Lactobacillus plantarum*	2.280	1590
**SL-15**	*Lactobacillus plantarum*	2.352	1590
**SL-16**	*Lactobacillus plantarum*	2.435	1590
**SL-17**	*Lactobacillus brevis*	2.449	1580
**SL-18**	*Lactobacillus plantarum*	2.396	1590
**SL-20**	*Lactobacillus plantarum*	2.327	1590
**SL-21**	*Lactobacillus plantarum*	2.381	1590
**SL-22**	*Lactobacillus plantarum*	2.566	1590
**SL-23**	*Lactobacillus plantarum*	2.371	1590
**SL-24**	*Lactobacillus plantarum*	2.440	1590
**SL-25**	*Lactobacillus brevis*	2.468	1580
**SL-26**	*Lactobacillus plantarum*	2.316	1590
**SL-27**	*Lactobacillus plantarum*	2.400	1590
**SL-28**	*Lactobacillus plantarum*	2.226	1590

Biotyper log(score) value: 3.0–2.3=highly probable species identification; 2.299 –2.000=definite genus identification, probable species identification; 1.999–1.700=probable genus identification; 1.699–0.0=not reliable identification. NCBI=The National Center for Biotechnology Information (*30*)

**Table S3:** MALDI-TOF mass spectrometry analysis of isolates of lactic acid bacteria grown on M17 medium obtained from sourdough of einkorn flour

**Isolate**	Similarity	Biotyper log(score)	NCBI identifier
**SL-29**	*Lactobacillus plantarum*	2.057	1590
**SL-30**	*Lactobacillus plantarum*	1.737	1590
**SL-31**	*Lactobacillus plantarum*	1.875	1590
**SL-32**	*Lactobacillus brevis*	2.369	1580
**SL-33**	*Lactobacillus brevis*	2.389	1580
**SL-34**	*Lactobacillus plantarum*	2.242	1590
**SL-35**	*Lactobacillus plantarum*	1.900	1590
**SL-36**	*Lactobacillus plantarum*	2.082	1590
**SL-37**	*Lactobacillus plantarum*	2.363	1590
**SL-38**	*Lactobacillus plantarum*	2.090	1590
**SL-39**	*Lactobacillus plantarum*	1.860	1590
**SL-40**	*Lactobacillus plantarum*	1.845	1590
**SL-42**	*Lactobacillus plantarum*	1.897	1590
**SL-43**	*Lactobacillus plantarum*	1.934	1590
**SL-44**	*Lactobacillus plantarum*	2.061	1590
**SL-45**	*Pediococcus pentosaceus*	2.114	1255
**SL-46**	*Lactobacillus plantarum*	1.942	1590
**SL-47**	*Lactobacillus plantarum*	1.871	1590
**SL-49**	*Lactobacillus plantarum*	2.033	1590
**SL-50**	*Lactobacillus plantarum*	2.092	1590
**SL-51**	*Lactobacillus paraplantarum*	1.702	60520
**SL-54**	*Lactobacillus paraplantarum*	1.988	60520
**SL-56**	*Lactobacillus plantarum*	1.988	1590
**SL-58**	*Lactobacillus paraplantarum*	2.027	60520
**SL-60**	*Enterococcus faecium*	1.914	1352
**SL-62**	*Lactobacillus plantarum*	1.922	1590
**SL-65**	*Lactobacillus paraplantarum*	2.018	60520
**SL-69**	*Lactobacillus paraplantarum*	1.758	60520
**SL-70**	*Enterococcus durans*	2.105	53345

Biotyper log(score) value: 3.0–2.3=highly probable species identification; 2.299 –2.000=definite genus identification, probable species identification; 1.999–1.700=probable genus identification; 1.699–0.0=not reliable identification. NCBI=The National Center for Biotechnology Information (*30*)

**Table S4:** Production of the exopolysaccharide (EPS) by isolates of lactic acid bacteria obtained from sourdough of einkorn flour

**Isolate**	**Similarity**	***γ(*EPS)/(g/L)**
**SL-1**	*Lactobacillus plantarum*	-
**SL-2**	*Lactobacillus plantarum*	1.1
**SL-3**	*Lactobacillus plantarum*	1.0
**SL-4**	*Lactobacillus plantarum*	1.0
**SL-5**	*Lactobacillus plantarum*	0.8
**SL-6**	*Lactobacillus plantarum*	1.1
**SL-7**	*Pediococcus pentosaceus*	0.7
**SL-8**	*Lactobacillus plantarum*	0.8
**SL-9**	*Lactobacillus plantarum*	0.8
**SL-10**	*Lactobacillus plantarum*	0.9
**SL-11**	*Lactobacillus plantarum*	1.1
**SL-12**	*Lactobacillus plantarum*	0.8
**SL-13**	*Lactobacillus plantarum*	1.0
**SL-14**	*Lactobacillus plantarum*	1.1
**SL-15**	*Lactobacillus plantarum*	1.1
**SL-16**	*Lactobacillus plantarum*	0.9
**SL-17**	*Lactobacillus brevis*	0.9
**SL-18**	*Lactobacillus plantarum*	1.0
**SL-20**	*Lactobacillus plantarum*	1.1
**SL-21**	*Lactobacillus plantarum*	1.1
**SL-22**	*Lactobacillus plantarum*	1.2
**SL-23**	*Lactobacillus plantarum*	1.0
**SL-24**	*Lactobacillus plantarum*	1.1
**SL-25**	*Lactobacillus brevis*	0.9
**SL-26**	*Lactobacillus plantarum*	1.1
**SL-27**	*Lactobacillus plantarum*	1.2
**SL-28**	*Lactobacillus plantarum*	1.2
**SL-29**	*Lactobacillus plantarum*	1.2
**SL-30**	*Lactobacillus plantarum*	0.9
**SL-31**	*Lactobacillus plantarum*	0.7
**SL-32**	*Lactobacillus brevis*	0.9
**SL-33**	*Lactobacillus brevis*	0.8
**SL-34**	*Lactobacillus plantarum*	0.9
**SL-35**	*Lactobacillus plantarum*	1.2
**SL-36**	*Lactobacillus plantarum*	1.3
**SL-37**	*Lactobacillus plantarum*	1.1
**SL-38**	*Lactobacillus plantarum*	2.4
**SL-39**	*Lactobacillus plantarum*	1.0
**SL-40**	*Lactobacillus plantarum*	1.9
**SL-42**	*Lactobacillus plantarum*	2.3
**SL-43**	*Lactobacillus plantarum*	0.6
**SL-44**	*Lactobacillus plantarum*	0.4
**SL-45**	*Pediococcus pentosaceus*	0.7
**SL-46**	*Lactobacillus plantarum*	0.7
**SL-47**	*Lactobacillus plantarum*	0.7
**SL-49**	*Lactobacillus plantarum*	0.7
**SL-50**	*Lactobacillus plantarum*	0.6
**SL-51**	*Lactobacillus paraplantarum*	0.6
**SL-54**	*Lactobacillus paraplantarum*	1.2
**SL-56**	*Lactobacillus plantarum*	1.0
**SL-58**	*Lactobacillus paraplantarum*	1.2
**SL-60**	*Enterococcus faecium*	0.8
**SL-62**	*Lactobacillus plantarum*	0.8
**SL-65**	*Lactobacillus paraplantarum*	1.2
**SL-69**	*Lactobacillus paraplantarum*	0.9
**SL-70**	*Enterococcus durans*	1.0
